# Anthelmintic Drug Resistance of Gastrointestinal Nematodes of Naturally Infected Goats in Haramaya, Ethiopia

**DOI:** 10.1155/2022/4025902

**Published:** 2022-01-17

**Authors:** Anteneh Wondimu, Yehualashet Bayu

**Affiliations:** ^1^College of Agriculture and Natural Resource, Bonga University, P.O. Box 334, Bonga, Ethiopia; ^2^College of Veterinary Medicine, Haramaya University, P.O. Box 138, Dire Dawa, Ethiopia

## Abstract

Gastrointestinal parasites and their anthelmintic resistance are major constraints to goat production in Ethiopia. Experimental investigation by faecal egg count reduction test (FECRT) and larval cultures were used to assess the occurrence of anthelmintic resistance in naturally infected goats with gastrointestinal nematodes (GIN) in Haramaya, Ethiopia. One hundred goats with a minimum of 150 eggs per gram (EPG) count were selected and randomly divided into five groups, 20 goats in each group, four treated and one untreated group. The result of the faecal egg count reduction test percentage (FECRT%) and the lower 95% confidence limit showed the presence of anthelmintic resistance for all tested drugs except tetramisole. FECRT% and lower 95% confidence limit were 69.9% and 36.9 for albendazole, 84.3% and 66.1 for tetraclozan, 95.7% and 87.4 for tetramisole, and 71.1% and 38.2 for ivermectin, respectively. *Trichostrongylus*, *Teladorsagia*, and *Haemonchus* showed anthelmintic resistance for tested drugs. Coproculture from different treatment groups revealed *Trichostrongylus* (69.2% in ivermectin and 59.6% in albendazole) were the predominant nematode followed by *Teladorsagia* (21.9% in albendazole and 14.7% in ivermectin). In tetraclozan treatment group, *Trichostrongylus* (42%) and *Teladorsagia* (41.3%) were comparable, followed by *Haemonchus* (13%). In group treated with tetramisole, *Teladorsagia* (54.3%) were the most frequently detected nematode followed by *Trichostrongylus* (25.7%) and *Haemonchus* (11.4%). Therefore, this study demonstrated the presence of multidrug resistant nematodes that may limit the productivity of goats. Moreover, further studies covering wider areas of Ethiopia and mechanisms of nematode resistance need to be studied in the future.

## 1. Introduction

Gastrointestinal (GI) parasites are a worldwide problem which reduces productivity of livestock in many countries. The impact is greater in sub-Saharan Africa including in Ethiopia due to the availability of a wide range of agro-ecological factors suitable for diversified host and parasite species [1–4]. The severity of helminthes parasites varies considerably depending on prevalence, genera, species involved, and local environmental such as humidity, temperature, and rainfall [5]. GI nematodes are the major causes of morbidity and mortality in goats. The death of the affected goat is mainly due to parasitic gastroenteritis [6]. Several studies also confirmed a widespread prevalence of sheep and goat nematodes in different parts of Ethiopia. For example, 69.01% of sheep and goats harbor one or more genera of nematodes [7]. Study in eastern part of Ethiopia stated the prevalence of nematodes in sheep and goats with *Haemonchus contortus* being the most prevalent followed by *Trichostrongylus* [8]. Another study in south west Ethiopia reported that 54.1% of sheep and goats were positive for GI parasite eggs [9].

Resistance of GI parasites to currently available anthelmintics has been reported worldwide. Different researchers also confirmed the occurrence of anthelmintic resistance (AR) to commonly used drugs. The problem associated with increased number of anthelmintic resistant parasites is becoming a major worldwide constrains in livestock production and hence need to detect and monitor resistance nematodes [10, 11]. The phenomenon of AR is spread in many countries with differences in prevalence and some data showed that GI nematodes develop anthelmintic resistance more rapidly in goats [12].

In Ethiopia, anthelmintic drugs commonly used for management of livestock GI nematodes fall under three families, including Bezimidazoles (albendazole and triclabendazole), imidazothiazoles (tetramisole and levamisole), and macrocyclic lactones (ivermectin). Misuse of these anthelmintic drugs has failed to decrease livestock GI parasite infection; but instead, led to the development of anthelmintic resistant nematodes in different agro-ecological zones of Ethiopia [3, 13, 14]. Failure to identify anthelmintic resistant parasites will also incur severe production consequences due to the impact of parasitic gastroenteritis [15].

Evidence of anthelmintic failure indicates the likelihood of suboptimal worm control, and hence, it is important to understand the geographic spread and severity of resistance for appropriate nematode control. Moreover, considering the narrow range of available drugs and slow rate of new drug development, AR presents an alarming global threat demanding vigilant monitoring and management. It is therefore considered likely that AR of GI nematodes of goats are present in Ethiopia, but little information regarding the prevalence and species of nematode resistance to drugs is available. Therefore, the objective of this paper is to assess anthelmintic resistance of GI nematodes of goats to commonly used anthelmintic drugs.

## 2. Material and Methods

### 2.1. Study Area

The study was conducted in Haramaya district, which is located approximately 510 km east of Addis Ababa, capital of Ethiopia. The estimated animal population is about 63,723 cattle, 79,950 sheep, and 120,350 goats. Topographically, it is situated at altitudes of 1600 to 2100 m above sea level, which puts the area into the category of a highland. Haramaya is located 9° 24′ N 42° 01′ E at an altitude of 1950 meters above sea level. The surrounding farming areas are semiarid. The mean annual rainfall received in the district ranges from 600 to 1260 mm. The district is representative of a subhumid midaltitude agro-climatic zone. The relative humidity varies between 60 and 80%. The minimum and maximum annual temperatures range from 6C to 12C and 17C to 25C, respectively [16].

### 2.2. Study Animals

Apparently, healthy goats maintained in Haramaya University (HU) goat farm were used to identify the GI nematodes of goats and their anthelmintic resistance (AR) status to commonly used anthelmintic drugs. A HU goat flock of approximately 150 heads of different breeds (Abergel, Somale, Cross breed, and Hararghe highlander), both sexes, different age, and weight groups was used. Each goat was individually marked with an ear tag before the start of the experiment. The goats were allowed to graze at Haramaya University pasture land during the daytime. In addition, supplemental hay and concentrate were provided during the dry season. Approximately 20 to 30 goats were bedding in groups for night shade, and house cleaning was done every day during the morning after goats were released for grazing. Sick goats and goats with newborns were also kept in a separate room for special treatments.

### 2.3. Determination of EPG

Before and after treatment of anthelmintic drugs, around 15 g of faecal samples were collected directly from the rectum of each goat early morning before allowed for grazing. Samples were placed in labeled polythene bags and immediately transported to the Haramaya University Animal Science Parasitology Laboratory for examination. Faecal samples were examined for helminth eggs using a modified McMaster technique in accordance with Urquhart et al. [17]. In each case, 3 g of faeces were mixed in 42 ml of saturated salt solution, and the number of nematode eggs per gram of faces (EPG) was obtained by multiplying the number of nematode eggs counted in two squares of the McMaster slide by a dilution factor of 50. The parasitic burden of the animals was evaluated based on EPG and goats with EPG count greater than 150 counts were included in the research and then grouped randomly into five treatment groups; four groups received drug treatments, and one group left untreated control. At ten-day intervals, faecal sample was collected from the goats, and immediately, laboratory analysis was done to determine the number of the parasite eggs (EPG) as described above, and anthelmintic resistance was evaluated based on the standard guideline.

### 2.4. Faecal Culture and Larval Recovery

Ova culture was performed on the unexploited faecal materials after EPG determination to obtain larval stages, as described by Van Wyk and Mayhew [18]. Faecal samples containing parasitic eggs were finely crushed with a pestle and mortar and were placed in a petri dish and incubated at room temperature (approximately 22–27°C) for 10 days and subjected to Baermann technique. Centrifuge tubes were filled to two thirds and spin at 1500 rpm for 5 minutes in an electrically powered centrifuge to concentrate the larvae to the bottom [19]. The larvae were recovered and pooled together after decantation of the supernatant fluid. A drop of lugol's iodine added to kill recovered larvae, then a drop of sample placed in microscope slide and cover-slip was applied, then the nematode larvae were identified to genera level and quantified based on morphology described by Van Wyk et al. [20] and John [21]. Where possible, 100 larvae were identified and counted for each experimental group of animals [21].

### 2.5. Experimental Design

The study goats were divided into five treatment groups, four treated and one untreated groups; ideally, 20 goats were included in each group and naturally exposed to GI nematodes. Each goat was weighed to give the correct dose of the anthelmintic drug. The drug given to animals was also based on manufacturer's recommendation. On day 0, faecal sample was collected from each goat enrolled in the study. Subsequently, faecal sample was collected 10 days after treatment. Larval cultures were also conducted pre- and posttreatment to determine specific nematode genera involved, to identify AR within specific parasitic genera. AR was assessed based on FECRT% explained by Coles et al. [22] in which AR occurs when FECRT% is less than 95%, and the lower 95% confidence level is lower than 90%. If only one of these was fulfilled, there was suspected AR. The sources of anthelmintic drugs were Chongqing Fangton Animal Pharmaceutical Co. Ltd, China (albendazole) from Ashish Life Science Pvt Limited India (tetramisole and tetraclozan) and Shenyang Sunvictor Pharmaceutical Co. Ltd. China (ivermectin). These drugs were chosen because of their availability and commonly used in the country.

Dosage was given as per manufacturer recommendations and considering the heaviest weight of goat in each group. Albendazole, tetramisole, and tetraclozan were given at the dosage rate of 7.5 mg/kg body weight orally while ivermectin injected subcutaneously at the dosage rate of 200 *μ*g/kg body weight.

### 2.6. Faecal Egg Count Reduction Test (FECRT)

FECRT was done based on reduction of the number of eggs per gram of faeces (EPG) by more than 95 percent measured ten days after treatment, the presence of resistance occurs if the reduction is less than 95%, and the lower confidence limit is less than 90%. The EPG count of goats which exceed 150 to 200 in the pretreatment group was included in the research [23]. The number of EPG from experimental groups before administration of drugs were done by determination of the mean level of egg excretion across animals (mean predrug administration (preDA) FEC), then after the administration of the drug (postDA FEC) as described above for the preDA FEC. Subsequently, the FECRT was calculated using the Coles et al.'s [22] method.

(1) $$\begin{array}{c} \text{FECRT }\left(\%\right)=100\;\left(1-\left[\frac{T_{2}}{C_{2}}\right]\right). \end{array}$$

where

*T*_2_ arithmetic mean FEC after treatment,

*C*_2_ arithmetic mean FEC control group at day 10.

Based on Coles et al.'s [22] method, resistance to an anthelmintic was considered to be present if the percentage reduction in egg count was less than 95%, and the lower 95% confidence limit is less than 90. But as most natural infections include a mixture of species, therefore, third stage larvae cultured from pretreatment and posttreatment groups were separately assessed to identify resistant nematodes [23].

### 2.7. Data Analysis

Anthelmintics efficacy was evaluated based on the reduction in faecal egg count and percentage of larvae found in the cultures. Calculation of the arithmetic mean, percentage of reduction, and 95% upper and lower confidence limits were conducted according to the procedures described by Coles et al. [22]. Resistance was declared when the percentage of reduction was less than 95%, and the 95% lower confidence limit was less than 90%. If only one of the two criteria was met, resistance was suspected.

## 3. Results

The result of EPG count showed that the minimum and maximum number of EPG count in pretreatment was 200 to 4600, respectively. Mean FEC before treatment for different drugs was from lower 690.5 to higher 1295.5, and the mean FEC post treatment ranges from 36.4 to 240.6. The result revealed FECRT% (69.9%, 84.3%, 95.7%, and 71.1%) and lower 95% confidence limit (36.9, 66.1, 87.4, and 38.2) for albendazole, tetraclozan, tetramisole, and ivermectin, respectively. The number showed FECRT% less than 95% and the lower 95% confidence level lower than 90% and demonstrated the presence of anthelmintic resistance (Table 1).

The dominant nematodes identified in pretreatment groups showed 66.2%, 57.6%, 56.4%, and 39.4% of *Trichostrongylus* in ivermectin, albendazole, tetramisole, and tetraclozan treatment group, respectively (Table 2). The result after treatment showed *Trichostrongylus* (69.2% in ivermectin and 59.6% in albendazole) as the predominant nematode followed by *Teladorsagia* (21.9% in albendazole and 14.7% in ivermectin). In the tetraclozan-treated group, 42% of *Trichostrongylus* and 41.3% of *Teladorsagia* were comparable followed by 13% of *Haemonchus.* FECRT revealed suspected AR was recorded to tetramisole, and a relatively lower number of parasitic counts were seen. The predominant nematodes identified were *Teladorsagia* 54.3% and *Trichostrongylus* 25.7% (Table 3).

## 4. Discussion

Anthelmintic resistance (AR) has been a global issue in the small ruminant industry during the past few decades [24]. Many parasites of veterinary importance have genetic features that favor the development of AR. Resistance by different species of nematodes to all major groups of anthelmintic drugs has been reported worldwide [11, 25, 26].

The present study also revealed the presence of multidrug resistance by different GI nematodes predominantly by *Trichostrongylus*, *Teladorsagia*, and *Haemonchus*. Comparable findings were reported in different parts of the world. In Uganda, 58%, 52%, and 38% AR prevalence in goat farms were detected for ivermectin, levamisole, and albendazole, respectively [27]. According to study by Crook et al. [28], *H. contortus, Trichostrongylus colubriformis*, and *Teladorsagia cirumcincta* developed resistance against levamisole and oxfendazole. Multidrug resistance was also recorded by *H. contortus* in sheep and goat. In addition, AR of *Teladorsagia/Trichostrongylus* against Benzimidazole was also found in Norway goats [29].

Factors like illegal marketing of drugs by nonanimal health professionals and purchasing of drugs by self-experience possibly allow inappropriate dosing or misuse that ends with survival of heterozygous resistant nematodes [30–32]. In addition, goats have a higher metabolic rate and require higher dose rates of drugs than sheep. This may explain the fact that AR is of greater concern in goats than in sheep [24, 29, 33]. The development of significant levels of resistance may also be due to the strong reliance on the same class of anthelmintic drugs without anthelmintic class rotation and lack of anthelmintic efficacy test [34, 35]. GI nematode composition observed in our study also implies that different nematode species may be more prone to the development of resistance to particular classes of anthelmintics.

Except tetramisole, the current study reported the presence of AR to all tested drugs (ivermectin, albendazole, and tetraclozan). Likewise, researchers from different parts of the world reported the occurrence of AR to this class of drugs. For example, study from India stated that most of the GI nematodes were found to have some degree of resistance against albendazole, levamisole, and ivermectin used in goats [36]. Another study in Italy revealed 40% and 20% of the goats flocks had resistant GI nematodes for benzimidazoles and ivermectin, respectively [6]. In addition, Adediran and Uwalaka [37] reported that GI nematodes of goats showed low resistance to ivermectin and levamisole but susceptibility to albendazole. Similar studies in the same study area and species of animal conducted by Sissay et al. [23] reported contrary results, i.e., FECRT% of tetramisole, albendazole, and ivermectin showed high levels of efficacy. The difference may explain the development of drug-resistant parasitic nematodes through time.

In the present study, *Trichostrongylus* were the dominant nematodes which developed resistance for albendazole and ivermectin followed by *Teladorsagia* and *Haemonchus*, while *Teladorsagia* were the dominant nematode developed suspected resistance for tetramisole followed by *Trichostrongylus* and *Haemonchus*; but in case of tetraclozan, *Trichostrongylus* and *Teladorsagia* showed almost equal percentage of resistance (Table 3). Comparable finding was recorded elsewhere in Ethiopia reported by Aga et al. [38] who stated a suspected resistance against albendazole by *H. contortus* and *Trichostrongylus*, but contrary to this study result, albendazole and tetramisole were found to possess a 100% efficacy against Ogaden isolate of *H. contortus*[39]; tetraclozan and ivermectin also demonstrated high efficacy against all nematode genera isolated on the farms [38]. Another study conducted by Sheferaw et al. [3] indicated that among the drugs used for treatments of nematodes, resistance to albendazole was suspected. Bersissa et al. [40] also recorded that albendazole and ivermectin were effective treatment for *Trichuris* and *Strongylus*, whereas tetramisole showed low efficacy, which partly agreed with low efficacy of tetramisole but disagreed with 100% efficacy of albendazole with the current report. The predominance of *Trichostrongylus* in the current study may be associated with Hypobiosis of other nematodes such as *Haemonchus* as the experiment done during the dry season of the year.

FECRT result showed the presence of AR to albendazole, tetraclozan, and ivermectin and suspected AR to tetramisole. The development of variable degrees of resistance among different species of GI nematodes has been reported for all the major groups of anthelmintic drugs by different scholars [37, 41, 42]. Resistance to anthelmintics has become a major problem in veterinary medicine and threatens both agricultural production and animal welfare, and there is increasing concern that drug resistance could arise in nematode parasites in humans. Thus, serious attention needs to be there to AR and inculcates other possible nematode control methods, which include management practices.

## 5. Conclusions

The current study demonstrated the presence of multidrug-resistant parasitic nematodes against albendazole, tetraclozan, and ivermectin. In addition, suspected resistance nematodes are also reported to tetramisole. *Trichostrongylus*, *Teladorsagia*, and *Haemonchus* showed anthelmintic resistance for all tested drugs. *Trichostrongylus* were the dominant nematode developed resistance for albendazole and ivermectin followed by *Teladorsagia* and *Haemonchus*, whereas *Teladorsagia* were the dominant nematode developed suspected resistance against tetramisole followed by *Trichostrongylus* and *Haemonchus*; *Trichostrongylus* and *Teladorsagia* showed almost equal percentage of resistance against tetraclozan. Therefore, further studies on anthelmintic resistance status of GIN covering wider areas of Ethiopia and on mechanisms of nematode resistance need to be studied in the future.

## Figures and Tables

**Table 1:** Anthelmintic resistance test based on percentage faecal egg count reduction test (FECRT%) according to Coles et al.'s [22] methods.

Drug	Ivermectin	Albendazole	Tetramisole	Tetraclozan	Control
No. of animals	20	20	20	20	20
Min and max EPG before Rx	200-4000	200-2200	200-4600	300-2200	50-1900
Min and max EPG after Rx	0-1050	0-1000	0-150	0-700	100-1000
MpreDA FEC (±SEM)	690.5 (±175.8)	781.2 (±172.7)	1295.5 (±492.9)	900 (±160.9)	850 (±343.4)
MpostDA FEC (±SEM)	230.9 (±130.6)	240.6 (±87.5)	36.4^a^ (±15.2)	125^a^ (±52.3)	800 (±89.5)
FECRT (%)	71.1	69.9	95.7	84.3	
95% CL	38.2-46.8	36.9-46.1	87.4-94.8	66.1-73.9	
Interpretation	Resistance	Resistance	Suspects	Resistance	

^a^Statistically different from EPG of control group day 10 (*p* < 0.05). MpreDA FEC: mean predrug administration FEC; MpostDA: mean postdrug administration FEC; FEC: fecal egg count; FECRT: fecal egg count reduction test; Min: minimum egg count; Max: maximum egg count; EPG: egg per gram of feces; CL: confidence level; SEM: standard error of mean.

**Table 2:** Percentage of nematode infective larvae identified based on the morphology of larvae (L3) from coproculture before administration of anthelmintic drugs.

Drug	Nematode larvae (L3)								
	Hae^a^	Tri^a^	Cha^a^	Oes^a^	Nem^a^	Str^a^	Tel^a^	Muc^a^	Coo^a^
Ivermectin	15.5	66.2	0	1.4	0.7	0	9.1	7	0
Albendazole	17.4	57.6	0	0	5.4	1	18.5	0	0
Tetramisole	8.3	56.4	0	2.5	1.9	2.5	18.6	6.4	3.2
Tetraclozan	17.8	39.4	2	2	0	0	24.6	14.4	0

^a^Values in pretreatments are percentages of nematode L3 larvae composition out of the 100 larvae counts. Tri: *Trichostrongylus*; Hae: *Haemonchus*; Cha: *Chabertia*; Oes: *Oesophagostomum*; Nem: *Nematodirus*; Str: *Strongyloides*; Tel: *Teladorsagia*; Mc: *Muellerius capillaris*; Coo: *Cooperia*.

**Table 3:** Percentage of different nematode infective larvae identified based on the morphology of larvae (L3) from coproculture of goats after administration of anthelmintic drugs.

Drug	Nematode larval (L3)								
	Hae^a^	Tri^a^	Cha^a^	Oes^a^	Nem^a^	Str^a^	Tel^a^	Muc^a^	Coo^a^
Ivermectin	4.9	69.2	0	0	0	0	14.7	2.8	8.4
Albendazole	10.5	59.6	0	0	1.7	0	21.9	5.2	0.9
Tetramisole	11.4	25.7	0	2.9	0	0	54.3	2.9	2.9
Tetraclozan	13	42	0	0	0	0	41.3	3.6	0

^a^Values in posttreatments are percentages of nematode L3 larvae composition out of the 100 larvae counts. Tri: *Trichostrongylus*; Hae: *Haemonchus*; Cha: *Chabertia*; Oes: *Oesophagostomum*; Nem: *Nematodirus*; Str: *Strongyloides*; Tel: *Teladorsagia*; Muc: *Muellerius capillaris*; Coo: *Cooperia*.

## Data Availability

The experimental data used to support the findings of this study may be released upon reasonable request to corresponding author.
